# Ultrasonographic and Computed Tomographic Diagnosis of Urachal Diverticulum Concurrent With Pyometra in a Dog

**DOI:** 10.1111/vru.70002

**Published:** 2025-01-10

**Authors:** Nicole Norena, Ryan B. Appleby, Alice Defarges, Lea Mehrkens, Jeff Biskup

**Affiliations:** ^1^ Department of Clinical Studies Ontario Veterinary College, University of Guelph Guelph Ontario Canada; ^2^ Capital City Specialty and Emergency Animal Hospital Ottawa Ontario Canada

**Keywords:** cystectomy, endometrial hyperplasia, pyometra, urachal anomaly, urachal diverticulum

## Abstract

A young, intact, female, American Bulldog was presented for hemorrhagic vaginal discharge. Anemia, thrombocytopenia, leukocytosis with neutrophilia, azotemia, and electrolyte disturbances were detected in the bloodwork. A urachal diverticulum with concurrent uterine distention was identified by ultrasonography and CT. The diverticulum was excised in surgery, and an ovariohysterectomy was performed. Histopathological examination confirmed an abscessed bladder wall with concurrent pyometra.

## 1 Signalment, History, and Clinical Findings

A 10‐month‐old, intact, female, American Bulldog presented for a 5‐day history of lethargy, inappetence, and hemorrhagic vaginal discharge. The dog went into her first heat 3 days prior to the clinical presentation while boarding at a training facility. Bloodwork provided in the referral history indicated a marked leukocytosis with neutrophilia, lymphocytosis, monocytosis, microcytic hyperchromic nonregenerative anemia, and thrombocytopenia. Serum biochemistry revealed marked azotemia and creatinine (too high to read) with hyperphosphatemia, severe hyperkalemia, and hypochloremia. Cytology of the vaginal discharge showed numerous rods and cocci. The dog was transferred to an emergency and referral hospital for further diagnosis and treatment.

## 2 Imaging, Diagnosis and Outcome

Point of care ultrasound identified scant free fluid and several large fluid‐filled structures throughout the abdomen thought to reflect uterine horns. There were several large pieces of hyperechoic material, interpreted to likely reflect blood clots or purulent material, within a markedly distended bladder.

**FIGURE 1:**
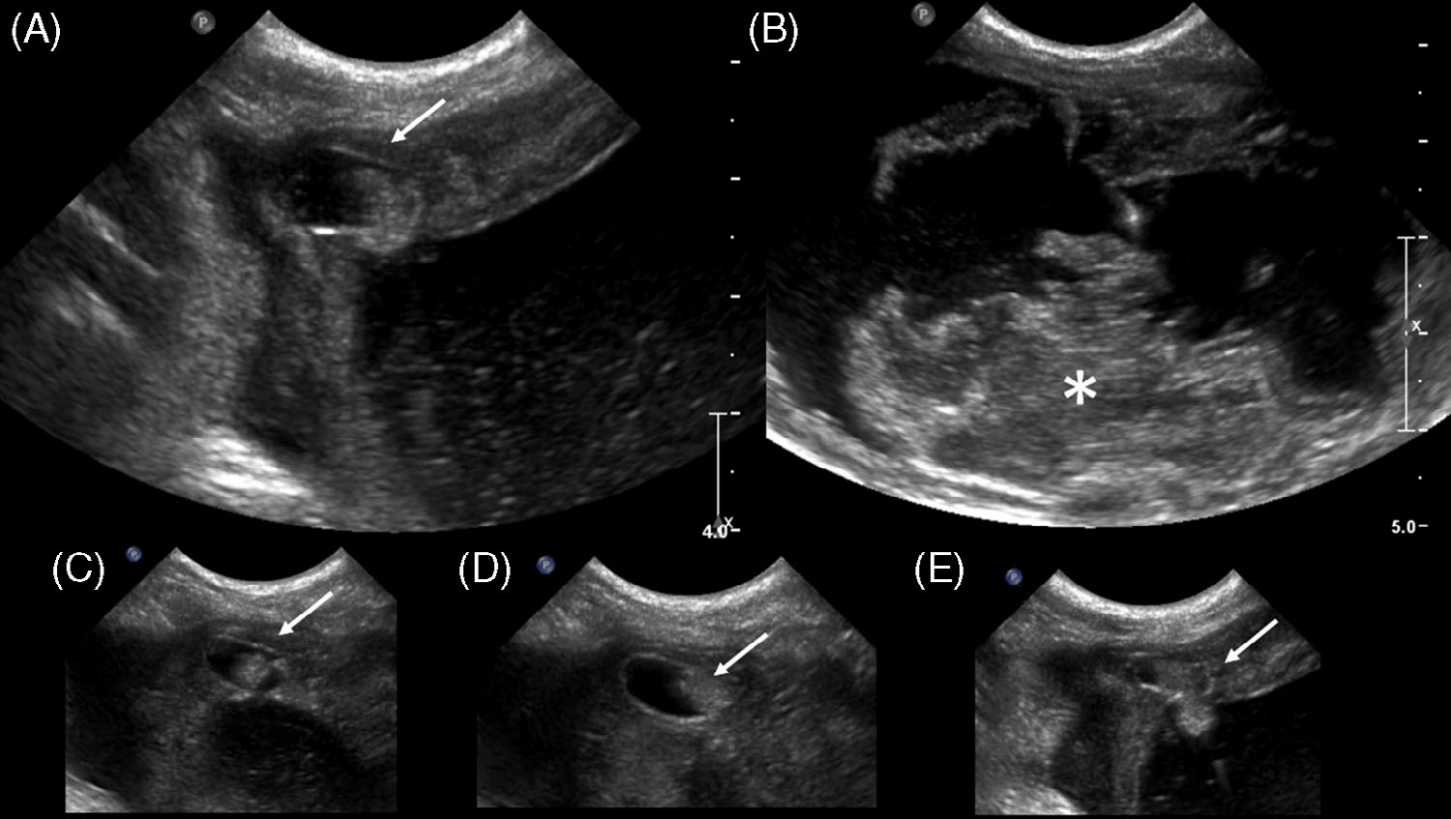
Longitudinal (A and B) and transverse (C–E) ultrasound images of the urinary bladder. An ovoid cavity approximately 1 cm mediolateral by 0.5 cm dorsoventral and craniocaudal is identified at the urinary bladder apex (arrow) which contains a combination of anechoic fluid and echogenic material. This cavity communicates with the bladder lumen and upon light ballottement of the bladder.

Follow‐up with a full abdominal ultrasound (Phillips IU22, 4–9 MHz convex probe and 5–12 MHz linear probe, small animal abdominal preset) revealed a markedly distended urinary bladder (UB) with a thickened wall and heterogeneous luminal content (Figure 1B). Markedly echogenic urine and heterogeneous amorphous pieces of tissue extended into the lumen of the bladder. At the apex of the UB, a small concavity (Figure 1A,C–E) containing anechoic fluid as well as echogenic material was identified. This cavity communicated with the bladder lumen (Figure 1E), and the echogenic material could be seen crossing the opening. The bladder wall surrounding the lesion was thickened up to 0.5 cm. The left uterine horn was distended up to 2 cm in diameter and contained echogenic fluid. The cervix was enlarged, 1.3 cm dorsoventral by 3.7 cm craniocaudal, and compressed the urethra at the level of the pelvic inlet. Partial urethral obstruction was suspected secondary to a mass effect caused by the enlarged cervix or the extension of echogenic bladder content plugging the urethra based on the degree of bladder distension and persistence over time. Mild bilateral pyelectasia was noted. The ultrasound imaging diagnosis suggested a complex congenital anomaly, including the urachal diverticulum with possible communication to the cervix. A neoplasm, a pyometra, or some combination thereof was considered possible. Given the unusual findings, a contrast‐enhanced CT was pursued to further characterize the lower urinary tract.

**FIGURE 2:**
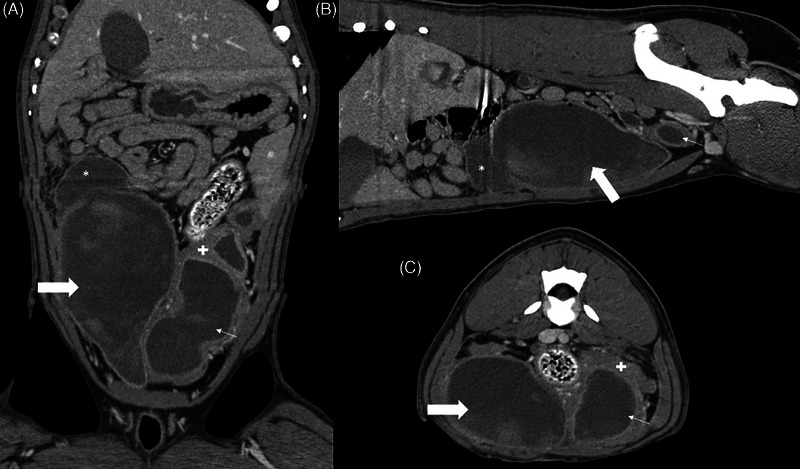
Dorsal (A), sagittal (B), and transverse (C) CT images postcontrast medium administration centered at the level of the urinary bladder and uterus. At the apex of the bladder is a crescent‐shaped‐fluid‐filled cavity (*), increased in size from the ultrasound examination (Figure 1) which communicates with the urinary bladder. The bladder (thick arrow) contains some relatively hyperattenuating material, similar in character to the left uterine horn (thin arrow). The uterine wall is moderately thick (+).

CT was performed using a GE Brightspeed 16‐slice scanner with images acquired in 0.6 mm slices and standard/soft tissue reconstruction. Images were available for both pre‐ and postiodinated contrast medium administration (2 mg/kg, Omnipaque 240 mg/mL). Three postcontrast imaging series were acquired immediately, at a 1‐ and 5 min delay, respectively. CT scan revealed a large fluid‐filled cystic mass located on the ventral aspect of the uterine body, occupying most of the pelvic canal dorsal to the urethra (Figure 2). The mass had a luminal content similar to that of the UB, and gas was found near the apex of the mass dorsally. The wall was undulating and irregular, with hyperattenuating material in the lumen. The mass extended to the left and thus contacted the left uterine horn and ovary but was thought to remain separate based on its appearance. Bilateral ovarian cysts were identified. A Foley catheter occupied the urethra but did not extend into the UB. The UB was markedly distended with concurrent thickening of the bladder wall. The UB was cranially displaced from the pelvic inlet extending to the right. At the cranial ventral aspect of the apex of the UB, a urachal diverticulum composed of a comparatively thin wall was found (Figure 2). The luminal content of the UB was variably hyperattenuating. The size of the urachal anomaly was increased relative to the previous abdominal US. Bilateral but asymmetric pyelectasia (left: 3.5 mm, right: 1 mm) was noted, consistent with the previous US findings. Ureteral distention was identified, and the walls were moderately contrast‐enhancing. The left ureter was wrapped from dorsal to ventral around the cystic uterine mass before emptying into the UB at the trigone.

Differentials for the uterine mass included endometrial hyperplasia and uterine neoplasia. Concurrent pyometra was considered possible. The findings associated with the urinary bladder confirmed the presence of a urachal diverticulum and its luminal content, as well as the thickening of the remainder of the bladder wall, supported a diagnosis of cystitis.

An exploratory laparotomy was performed, which revealed a thickened bladder with purulent discharge draining from its apex. During the ventral cystotomy, a necrotic urachal diverticulum was confirmed upon entry. The apex of the bladder and necrotic diverticulum were removed during a partial cystectomy. The bladder was leak‐tested after closure. Additionally, a routine ovariohysterectomy was performed. A large firm area was palpated just distal to the cervix. The uterus was removed at the level of the proximal vagina to remove the mass lesion. There was no evidence of bleeding during the evaluation.

A portion of the bladder wall, diverticulum, uterus, and ovaries were submitted to IDEXX Laboratories‐(Markham, Ontario, Canada) for a histopathological evaluation. The final histopathological diagnosis was consistent with focal cystic endometrial hyperplasia with pyometra and ovarian cysts. The urinary bladder and diverticulum samples showed chronic inflammation with proliferative granulation tissue, prominent neutrophil infiltration, and necrosis. Special stains revealed the presence of Gram‐positive bacterial rods, consistent with an infectious etiology of the bladder wall lesion.

## 3 Discussion

To the authors’ knowledge, this is the first published report that describes a urachal diverticulum concurrent with a pyometra in a dog. Urachal anomalies have been described in small companion animals, large animals, and humans [1, 2, 3, 4, 5]. A recent study found the prevalence of UA in companion animals to be low (0.93% in cats and 0.18% in dogs) [1]. Similarly, in human studies, the reported prevalence of UA is approximately one in 5000 adults and even lower in infants [6]. As a result of the widespread use of cross‐sectional imaging, incidental UAs have been more frequently identified in humans and animals [7].

Urachal diverticula have been reported to have the highest prevalence in dogs compared with other UAs, with varying sizes and clinical signs reported [1, 8, 9]. A diagnosis can be confirmed using various imaging techniques (ultrasonography, CT, or contrast‐enhanced cystography); however, ultrasonography remains the most widely used since it is readily available and is noninvasive [8]. On ultrasound, a urachal diverticulum appears as an extraluminally protruding, fluid‐filled structure extending from the cranioventral aspect of the bladder. In people, in CT imaging urachal diverticula are often an incidental finding, characterized by a cystic lesion located just above the superior aspect of the bladder without an umbilical connection [10]. In the case presented here, a UA was detected incidentally during an abdominal ultrasound screening for suspected pyometra. Based on the degree of clinical signs and the numerous concurrent US abnormalities, a CT was recommended for further characterization. While CT added value in supporting the diagnosis and guiding surgery, a surgical exploration could have been pursued with ultrasound alone.

UAs are thought to predispose dogs to recurrent bacterial urinary tract infections (BUTI) and cystitis [9]. Pollakiuria, stranguria, hematuria, and urinary incontinence are among the most common clinical signs in dogs with a UA and concurrent BUTI [9]. Clinical signs of urachal diverticula can indicate an infection or neoplasm in the bladder, but they are often asymptomatic in both humans and animals [11, 12]. In this case, the dog presented with some clinical signs characteristic of a pyometra (lethargy, inappetence, and hemorrhagic vaginal discharge) but lacking polyuria and polydipsia. A study found that urinary tract infections were the second most common complication in bitches with pyometra [13]. Therefore, it is possible that the abscessed diverticulum was induced by a pyometra‐related complication. While the order of occurrence (urachal anomaly infection leading to uterine infection or uterine infection leading to bladder and urachal anomaly infection) cannot be determined in this case, the concurrent nature of the infection reflects a unique presentation. Acquired urinary bladder diverticula are rare in dogs; however, they can develop following a traumatic injury to the bladder or when an outflow obstruction occurs. Scheepens and L'Eplattenier [14]. described a suspected acquired urinary bladder diverticulum in a 6‐year‐old spayed female Chow Chow that was surgically repaired. Additionally, there are cases of acquired UAs that can be managed medically [15].

This case serves as a novel example of an abscessed urachal diverticulum with concurrent pyometra and is the first reported case in a dog. The imaging findings reflect a novel presentation that radiologists should be aware of and consider when faced with these rare findings.

## List of Author Contributions

### Category 1

(a) Conception and design: Norena, Appleby
(b) Acquisition of data: Defarges, Mehrkens, Biskup
(c) Analysis and interpretation of data: Norena, Appleby, Defarges, Mehrkens, Biskup

### Category 2

(a) Drafting the article: Norena
(b) Revising article for intellectual content: Norena, Appleby, Defarges, Mehrkens, Biskup

### Category 3

(a) Final approval of the completed article: Norena, Appleby, Defarges, Mehrkens, Biskup

## Conflict of Interest

The authors declare no conflict of interest.

## Data Accessibility Statement

None.

## Reporting Checklist

No reporting checklist was used

## Previous Publication or Presentation

No prior presentation or publication.
